# Stage-Specific COPII-Mediated Cargo Selectivity in African Trypanosomes

**DOI:** 10.1128/msphere.00188-22

**Published:** 2022-06-21

**Authors:** Mohamed Sharif, James D. Bangs

**Affiliations:** a Department of Biochemistry, Jacobs School of Medicine and Biomedical Sciences, University at Buffalo, Buffalo, New York, USA; b Department of Microbiology and Immunology, Jacobs School of Medicine and Biomedical Sciences, University at Buffalo, Buffalo, New York, USA; University of Texas Southwestern

**Keywords:** trypanosome, variant surface glycoprotein, COPII, glycosylphosphatidylinositol, ER exit

## Abstract

A hallmark of eukaryotic cells is the ability to form a secretory pathway connecting many intracellular compartments. In the early secretory pathway, coated protein complex II (COPII)-coated vesicles mediate the anterograde transport of newly synthesized secretory cargo from the endoplasmic reticulum to the Golgi apparatus. The COPII coat complex is comprised of an inner layer of Sec23/Sec24 heterodimers and an outer layer of Sec13/Sec31 heterotetramers. In African trypanosomes, there are two paralogues each of Sec23 and Sec24, that form obligate heterodimers (TbSec23.2/TbSec24.1, TbSec23.1/TbSec24.2). It is not known if these form distinct homotypic classes of vesicles or one heterotypic class, but it is known that TbSec23.2/TbSec24.1 specifically mediate forward trafficking of GPI-anchored proteins (GPI-APs) in bloodstream-form trypanosomes (BSF). Here, we showed that this selectivity was lost in insect procyclic stage parasites (PCF). All isoforms of TbSec23 and TbSec24 are essential in PCF parasites as judged by RNAi knockdowns. RNAi silencing of each subunit had equivalent effects on the trafficking of GPI-APs and p67, a transmembrane lysosomal protein. However, silencing of the TbSec23.2/TbSec24.1 had heterodimer had a significant impact on COPII mediated trafficking of soluble TbCatL from the ER to the lysosome. This finding suggests a model in which selectivity of COPII transport was altered between the BSF and PCF trypanosomes, possibly as an adaptation to a digenetic life cycle.

**IMPORTANCE** African trypanosomes synthesize dense surface coats composed of stage-specific glycosylphosphatidylinositol lipid anchored proteins. We previously defined specific machinery in bloodstream stage parasites that mediate the exit of these proteins from the endoplasmic reticulum. Here, we performed similar analyses in the procyclic insect stage and found significant differences in this process. These findings contribute to our understanding of secretory processes in this unusual eukaryotic model system.

## INTRODUCTION

The pathogenic protozoan parasite Trypanosoma brucei spp. is the causative agent of human African trypanosomiasis (HAT, sleeping sickness) in sub-Saharan Africa (1). In 2019, the estimated population at risk was 65 million individuals, and 992 new cases of HAT were reported (World Health Organization, www.who.int). It is assumed that these figures are underreported and many more are infected with HAT across Africa (2). While still clinically relevant, the parasite also causes disease in cattle known as nagana (3, 4), which leads to significant economic consequences in affected areas (5). The parasite has a digenetic life cycle alternating between the bloodstream form (BSF) in the mammalian host and several other forms in the tsetse fly vector (*Glossina* subsp.), including the procyclic form (PCF) found in the vector midgut (6). As such, these protozoan parasites encounter multiple hostile extracellular microenvironments (7–9) and, as an adaptation to their respective environments, both BSF and PCF stages express unique densely packed glycosylphosphatidylinositol (GPI)-anchored proteins as a surface coat (10, 11).

BSF trypanosomes express antigenically distinct variant surface glycoproteins (VSG). Accounting for approximately 10% of the total protein synthesized in the BSF stage, the expression of VSG is critical for the extracellular survival and pathogenesis of trypanosomes (12, 13). VSG, a homodimer, forms a monolayer covering the entire cell body and flagellum. These 5 × 10^6^ VSG dimers protect underlying invariant proteins from host immune responses. At any given time, trypanosomes express only one of the hundreds of nuclear-encoded *VSG* genes and switch VSG expression through a process known as antigenic variation, which is essential for prolonged infection. Hence, the efficient expression, biosynthesis, and transport of VSG proteins are critical to parasite survival and life cycle progression. When the tsetse fly ingests BSF trypanosomes, the parasite differentiates to PCF, simultaneously shedding the VSG coat and replacing it with procyclin, which is also GPI-anchored (14, 15). This procyclin surface coat enables the parasite to survive in the harsh proteolytic conditions of the fly midgut (8, 16). There are two classes of procyclin, EP and GPEET, uniquely characterized by internal dipeptide (EP) or pentapeptide (GPEET) repeats. Trypanosomes express three EP (EP1, EP2, EP3) isoforms and a single GPEET protein (17).

In comparison to VSG, procyclins are tethered to the cell surface by a distinct stage-specific GPI-anchor structure (11). Before exit from the endoplasmic reticulum (ER), the core glycan structure (one glucosamine and three mannoses), and terminal phosphoethanolamine of the GPI-anchor are common between BSF and PCF trypanosomes. However, the lipid configuration is different between the two stages. Nonetheless, in each stage, this structure acts as a forward trafficking signal for ER exit (18, 19). In BSF trypanosomes, loss of the GPI-anchor on VSG results in a significant delay in ER exit and subsequent misdirection in post-Golgi compartments from the cell surface to the lysosome. Conversely, the addition of a GPI-anchor to BiPN, a small globular bulk flow secretory reporter, results in accelerated trafficking from the ER (20, 21). Similar results were observed in PCF trypanosomes with ectopically expressed GPI-minus and GPI-plus VSG and BiPN reporters (21, 22). Collectively these data indicate that the GPI-anchor acts as a forward trafficking signal for secretory cargo in anterograde transport from the ER to the Golgi.

**FIG 1:**
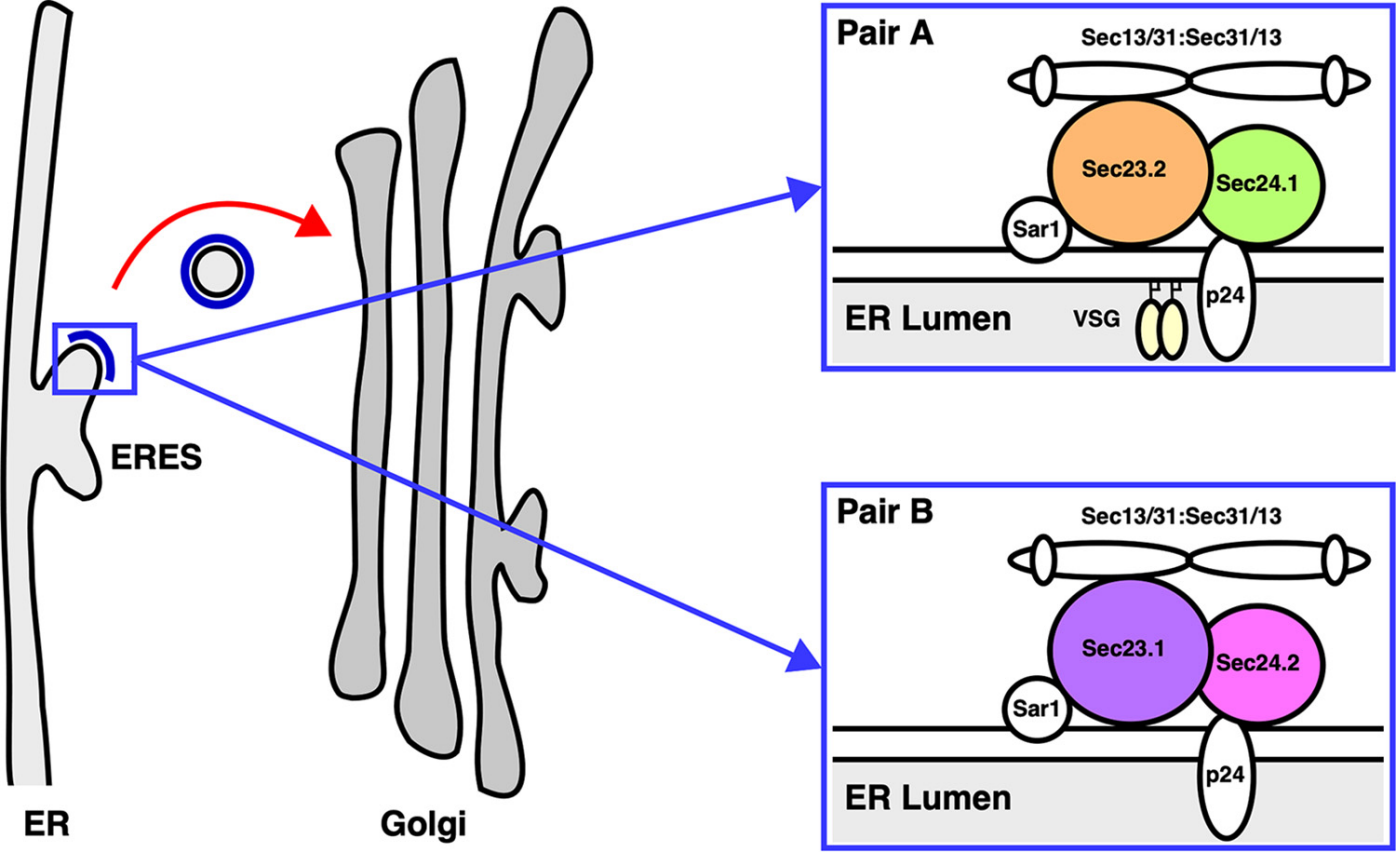
Trypanosome COPII machinery. Schematic of the early secretory pathway from the ERES to the Golgi apparatus. Anterograde transport (red arrow) of secretory cargo is facilitated by COPII vesicles comprised of outer heterotetrameric (Sec13/31:Sec31/13) and inner heterodimeric (Sec23/Sec24) layers (blue). The inner layer of COPII vesicles is comprised of obligate and specific heterodimers (Pair A: TbSec23.2/TbSec24.1 and/or Pair B: TbSec23.1/TbSec24.2). It is Pair A that in BSF trypanosomes selectively mediates ER exit of GPI-APs such as VSG dimers (yellow) via interactions with a cohort of p24 transmembrane adapter proteins called TbERPs.

Like most secretory cargoes in eukaryotes, GPI-anchored proteins (GPI-APs) are transported from the ER by coat protein complex II (COPII) vesicles that bud from ER exit site (ERES) (23) (Fig. 1). Sequentially, Sec12 (24), an ER-localized guanine nucleotide exchange factor, initiates COPII formation by inducing a GDP (GDP) to GTP (GTP) exchange on Sar1 (25, 26), a cytosolic GTPase. Sar1-GTP then undergoes a conformational change, embeds into the ER membrane, and recruits Sec23/Sec24 heterodimers via specific interaction with Sec23. The Sar1-Sec23/Sec24 “prebudding complex” is responsible for the capture of transmembrane cargo via interactions with Sec24 (27). Subsequently, after cargo capture, Sec13/Sec31 heterotetramers are recruited to the prebudding complex resulting in the deformation and budding of discrete COPII vesicles (28, 29). Unlike transmembrane secretory cargo, GPI-anchored and soluble proteins face the ER lumen and are topologically sequestered from the COPII machinery. Cohorts of p24 transmembrane adaptor proteins facilitate the interaction between the GPI-APs, as well as soluble cargo, and the COPII machinery (30, 31). Eukaryotic cells have multiple p24 genes: 11 in Arabidopsis thaliana (*A*. *thaliana*), 8 in Saccharomyces cerevisiae (S. cerevisiae), and 10 in mammalians (32–34). These p24 subunits assemble via lumenal coiled:coil interactions in a combinatorial manner to form higher-order oligomeric receptor complexes. The formation of p24 complexes is essential for receptor stability and recognition of cargo in the ER lumen and the COPII machinery in the cytoplasm.

There are two paralogues in trypanosomes for Sec23 (TbSec23.1 and TbSec23.2) and Sec24 (TbSec24.1 and TbSec24.2) (35). These COPII subunits form two obligate and specific pairs: TbSec23.2/TbSec24.1 (Pair A) and TbSec23.1/TbSec24.2 (Pair B) (Fig. 1). Interestingly, in RNAi silencing studies, Pair A selectively mediates ER exit of VSG in BSF trypanosomes. Loss of either TbSec23.2 or TbSec24.1 results in a 4 to 5-fold decrease in the rate of VSG transport. This selectivity is only for GPI-APs, as there was no effect of silencing on the trafficking of soluble or transmembrane cargo. Trypanosomes have eight p24 orthologues (TbERPs: T. brucei Emp24-related proteins), all expressed at the mRNA level in both BSF and PCF trypanosomes (21). However, at the protein level only TbERP1, 2, 3, and 8 are expressed in BSF trypanosomes, while only TbERP1, 2, 4, and 8 are expressed in PCF parasites. Independent RNAi silencing of expressed TbERPs in BSF trypanosomes indicates that p24 complexes mediate ER exit of GPI-anchored and soluble cargo.

While there are major differences in secretory protein trafficking between BSF and PCF trypanosomes, including pathways, cargo, GPI-anchor structure, and p24 adaptor transmembrane proteins, little is known of the role of COPII in GPI-dependent trafficking in PCF trypanosomes. To this aim, we have performed independent genetic characterization of each of the TbSec23 and TbSec24 subunits using RNA interference (RNAi) silencing along with transport assays for various secretory reporters. Our results suggest a loss of COPII-dependent selectivity for ER exit of GPI-anchored cargo and revealed a new selectivity in the transport of soluble secretory cargoes.

## RESULTS

### TbSec23 and TbSec24 are required in PCF trypanosomes.

**FIG 2:**
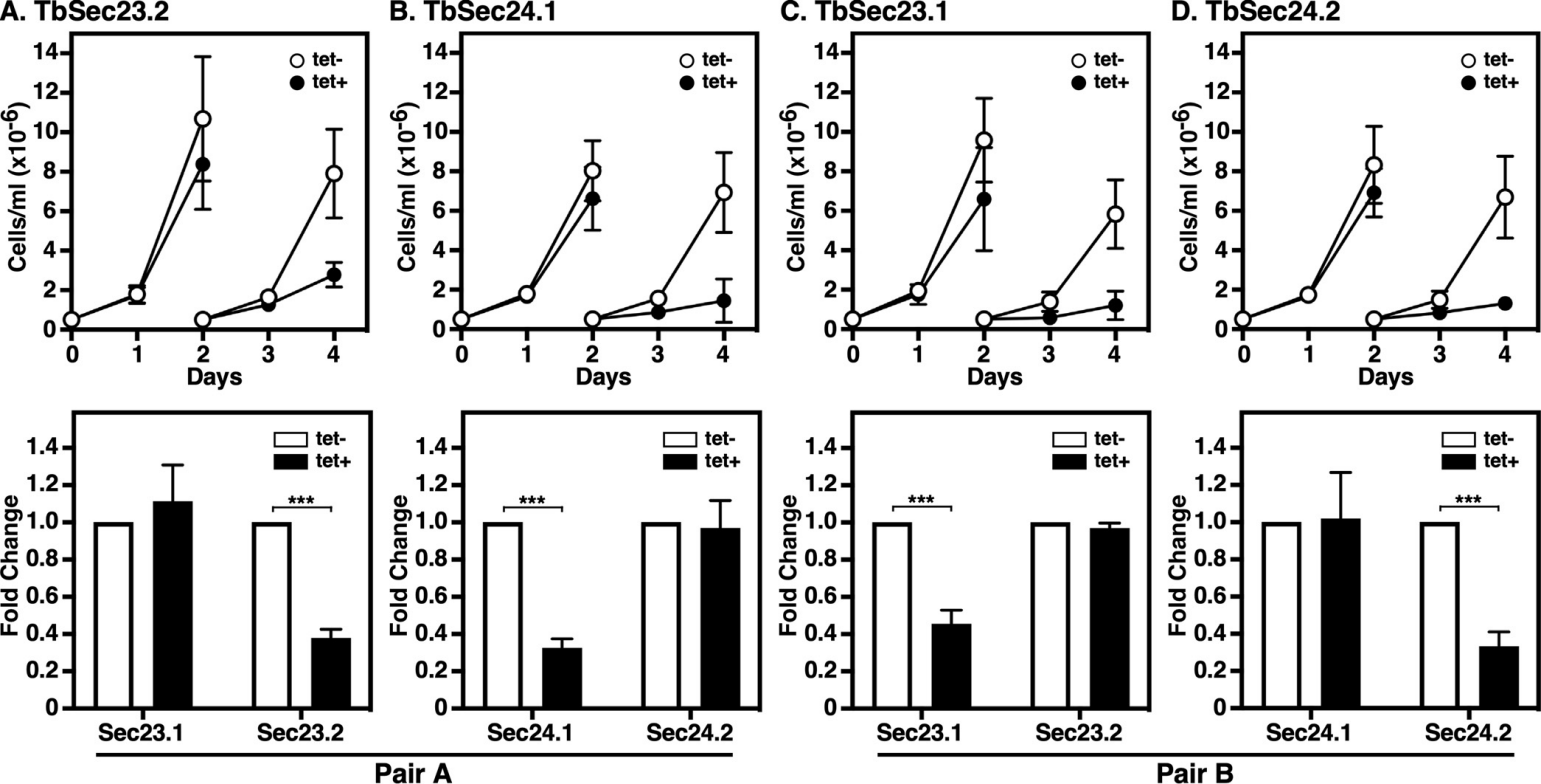
Silencing of TbSec23 and TbSec24. TbSec23.2 (A), TbSec24.1 (B), TbSec23.1 (C) and TbSec24.2 RNAi cell lines were cultured with (Tet^+^) and without (Tet^−^) tetracycline to initiate dsRNA production. Pair A and Pair B are indicated. (Top) Cells were seeded at 5 × 10^5^ cells/mL and counted daily. Every 2 days, cells were adjusted to the starting density to maintain log phase growth. (Bottom) mRNA levels in control and silenced cells were evaluated at 2 days postinduction using qRT-PCR. mRNA levels were normalized using the internal control, TbZFP3. Data are presented as the fold change from uninduced control. Both growth and knockdown efficiency assays were performed in triplicate and three biological replicates were conducted. The data are presented as mean ± SD. Significance was calculated using Student’s *t* test with ***, *P* ≤ 0.001.

Our previous work showed that RNAi silencing of each TbSec23 or TbSec24 paralogue in BSF trypanosomes was lethal but had minimal effect on the trafficking of soluble and transmembrane secretory proteins. However, silencing of TbSec23.2/TbSec24.1 (Pair A), but not TbSec23.1/TbSec24.2 (Pair B), specifically delayed the trafficking of GPI-anchored cargo from the ER (35). This raises the question of whether Pair A-specific GPI-dependent ER exit also occurs in PCF parasites. Conditional RNAi constructs targeting each of the TbSec23 or TbSec24 paralogues were introduced into tetracycline-responsive PCF cells, clones were selected, and genomic insertion of the RNAi construct was confirmed by PCR (Fig. S1B and C). In all cases, RNAi silencing in PCF trypanosomes resulted in growth arrest by day two (Fig. 2, top). However, in all four induced cell lines, cell growth recovered after day 6 (unpublished data), likely due to the loss of RNAi function. With respect to the TbSec24 depletions specifically, our results confirmed those of Demmel et al. (36) in PCF trypanosomes.

10.1128/msphere.00188-22.1 FIG S1 COPII Machinery and specific Sec23 or Sec24 RNAi cell lines. (A) Schematic of trypanosome COPII machinery with Pair A and Pair B color-coded: TbSec23.2 (orange), TbSec24.1 (green), TbSec23.1 (purple), and TbSec24.2 (pink). (B) Schematic of primer target locations for confirming specific insertion of RNAi constructs into cellular genomic DNA. p2T7Ti, a vector with dual promoters (red arrows), was used to clone the four RNAi constructs (sky-blue rectangle) to generate double-stranded RNAi. For confirmation of genomic insertion, the forward primer (gray) targeted a common upstream region on the inserted vector's backbone (dotted line) and the reverse primers targeted specific internal regions of the TbSec23/24 ORFs. All four reverse primers have the same colors as in schematic (A). (C) Genomic PCR results for each of the four RNAi cell lines. Each cell line was tested with each of the four specific reverse primers: TbSec23.2, TbSec24.1, TbSec23.1, and TbSec24.2 as indicated. In each case, specific DNA products were generated for each of the four RNAi cell lines as indicated. Collectively, these data confirm the specific insertion of the RNAi constructs into the cellular genomic DNA. Download FIG S1, PDF file, 0.07 MB. Copyright © 2022 Sharif and Bangs. 2022 Sharif and Bangs. https://creativecommons.org/licenses/by/4.0/ This content is distributed under the terms of the Creative Commons Attribution 4.0 International license.

All subsequent RNAi phenotypic analyses were done 2 days postinduction as gross cell morphology remained intact at this time point. In addition, immunofluorescence microscopy localizing BiP (37), an ER marker, and p67 (38), a lysosomal marker, indicate normal internal morphology (Fig. S2). Knockdown efficiency was also assessed on day 2 using quantitative real-time PCR (qRT-PCR) (Fig. 2, bottom). In all cases, statistically significant (*P* ≤ 0.001) depletion (~60%) of target mRNA was achieved. In each case, the knockdown was specific to the targeted TbSec subunits. Paralogous subunits were unaffected. Collectively, these data indicated that all TbSec23 and TbSec24 subunits are critical for normal growth in PCF trypanosomes.

10.1128/msphere.00188-22.2 FIG S2 Localization of BiP and p67 in TbSec23/24 knockdowns. TbSec23.1 (A), TbSec23.2 (B), TbSec24.1 (C), and TbSec24.2 (D) RNAi cell lines were cultured with (Tet^+^) or without (Tet^−^) tetracycline to initiate specific dsRNA production for 48 h. Cells were fixed and immunostained with anti-BiP (green) and anti-p67 (red) to localize the ER and lysosome (l), respectively. The nucleus (n) and kinetoplast (k) were stained with DAPI. Immunofluorescence microscopy was performed, and representative 3-channel summed-stack projections are presented. Cell outlines were drawn from matching DIC Images. White bar indicates 2 μm. Typical staining patterns in all four RNAi cell lines were observed for BiP and p67, indicating normal internal morphology. Download FIG S2, PDF file, 0.9 MB. Copyright © 2022 Sharif and Bangs. 2022 Sharif and Bangs. https://creativecommons.org/licenses/by/4.0/ This content is distributed under the terms of the Creative Commons Attribution 4.0 International license.

### TbSec23s localize to the ERES in PCF trypanosomes.

**FIG 3:**
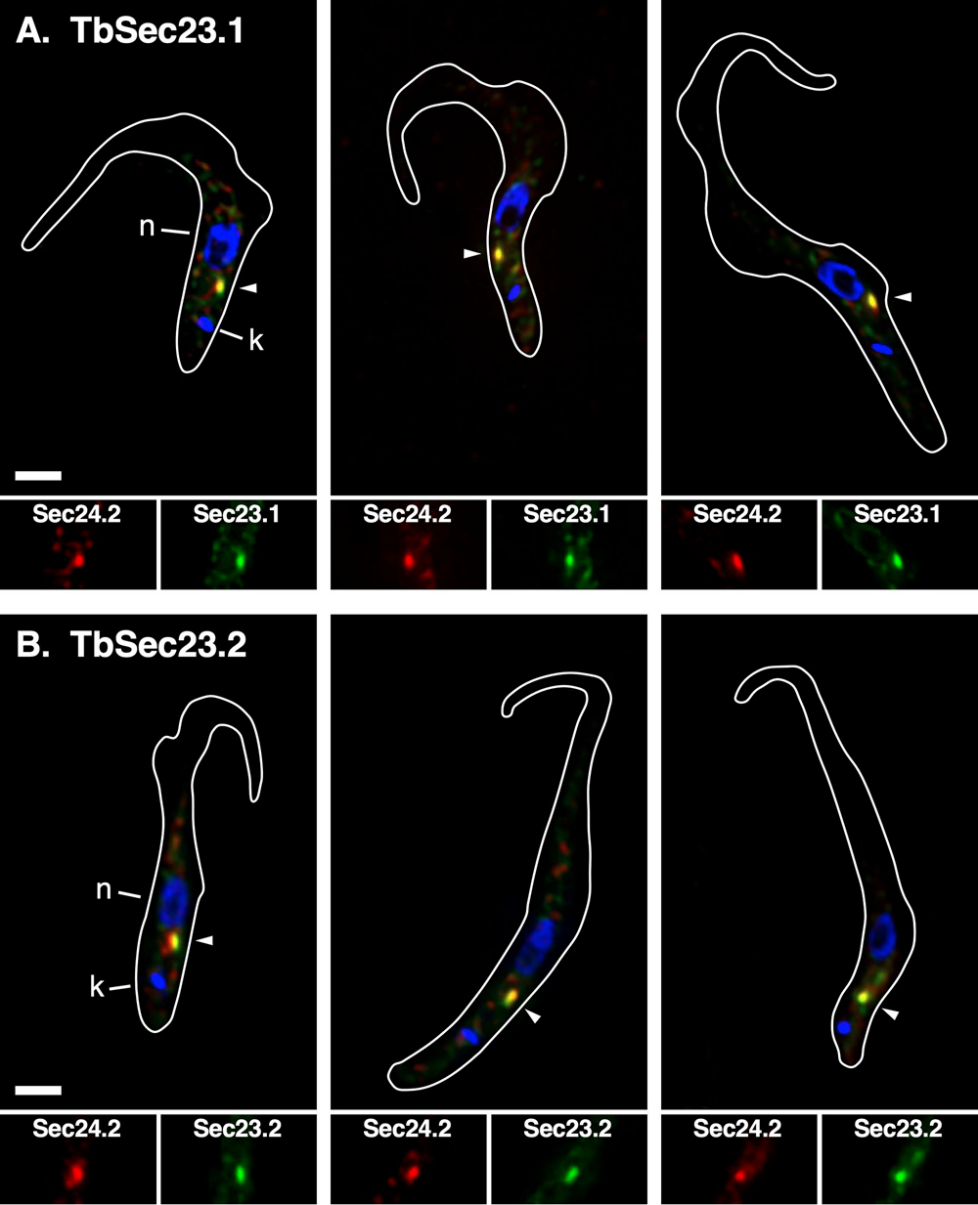
Localization of TbSec23 subunits. TbSec23.1:HA (A) and TbSec23.2:HA (B) were independently cloned into the TbSec24.2:Ty host cell line and the double HA/Ty-tagged cell lines were immunostained with anti-Ty (red; TbSec24.2) and anti-HA (green; TbSec23.1 or TbSec23.2). 3-channel summed stack projection images are presented (top). Location of the nucleus (blue; n), kinetoplast (blue; k), and ERES (arrowhead) are indicated. Matched single-channel red (TbSec24.1) and green (TbSec23.2 or TbSec23.1) images of the ERES region are presented (bottom). Cell outlines are drawn from matched DIC images. White bar indicates 2 μm.

Previously we found that all four TbSec23/TbSec24 subunits colocalized to the two ER exit sites (ERES) in interphase BSF trypanosomes (35). Furthermore, both TbSec24 subunits had been localized to the single ERES in PCF cells (36). To localize TbSec23 subunits in PCF trypanosomes we used a TbSec24.2:Ty host cell line as an ERES marker. Each *TbSec23* gene was independently HA-tagged to generate TbSec23.2:HA/TbSec24.2:Ty and TbSec23.1:HA/TbSec24.2:Ty cell lines. Immunoblotting confirmed the expression of tagged proteins of the expected sizes (Fig. S3). Immunofluorescence was performed using anti-HA and anti-Ty (Fig. 3). As expected, each TbSec23:HA reporter colocalized strongly with TbSec24.2:Ty at the known single ERES between the nucleus and kinetoplast, confirming that PCF trypanosomes have a single ERES where all inner COPII subunits colocalize.

10.1128/msphere.00188-22.3 FIG S3 Epitope tagging of TbSec23.1 and TbSec23.2. Whole-cell extracts of the dual epitope-tagged TbSec23.1HA/TbSec24.2:Ty and TbSec23.2HA/TbSec24.2:Ty cell lines were subjected to simultaneous immunoblotting with rabbit anti-HA and monoclonal anti-Ty. In each cell line, Sec24.1:Ty of the appropriate molecular mass was observed. This reporter is derived from the parental cell line (not shown). As appropriate, tagged Sec23.1:HA (lane 1) and Sec23.2:HA (lane 2) were observed. These results confirm the expression of each tagged reporter. Download FIG S3, PDF file, 0.04 MB. Copyright © 2022 Sharif and Bangs. 2022 Sharif and Bangs. https://creativecommons.org/licenses/by/4.0/ This content is distributed under the terms of the Creative Commons Attribution 4.0 International license.

### ER exit of GPI-anchored cargo is not COPII-selective in PCF trypanosomes.

We have shown that GPI-dependent ER exit in BSF trypanosomes is mediated selectively by Pair A (35). To investigate this process in PCF cells, the native surface coat protein, procyclin, is not a useful reporter because it does not contain methionine or cysteine for radiolabeling, and there is no convenient assay for ER exit/surface arrival. However, we have used the ectopic expression of BSF VSG as a surrogate to monitor the trafficking of GPI-APs in PCF cells (18, 22). Similar to BSF cells, VSG was GPI-anchored, dimerized, and exported to the cell surface in a GPI-dependent manner. However, in PCF trypanosomes VSG was cleaved and released upon arrival at the cell surface by a resident stage-specific zinc metalloprotease, MSP-B (22, 39), which served as the basis for a quantitative pulse/chase transport assay.

**FIG 4:**
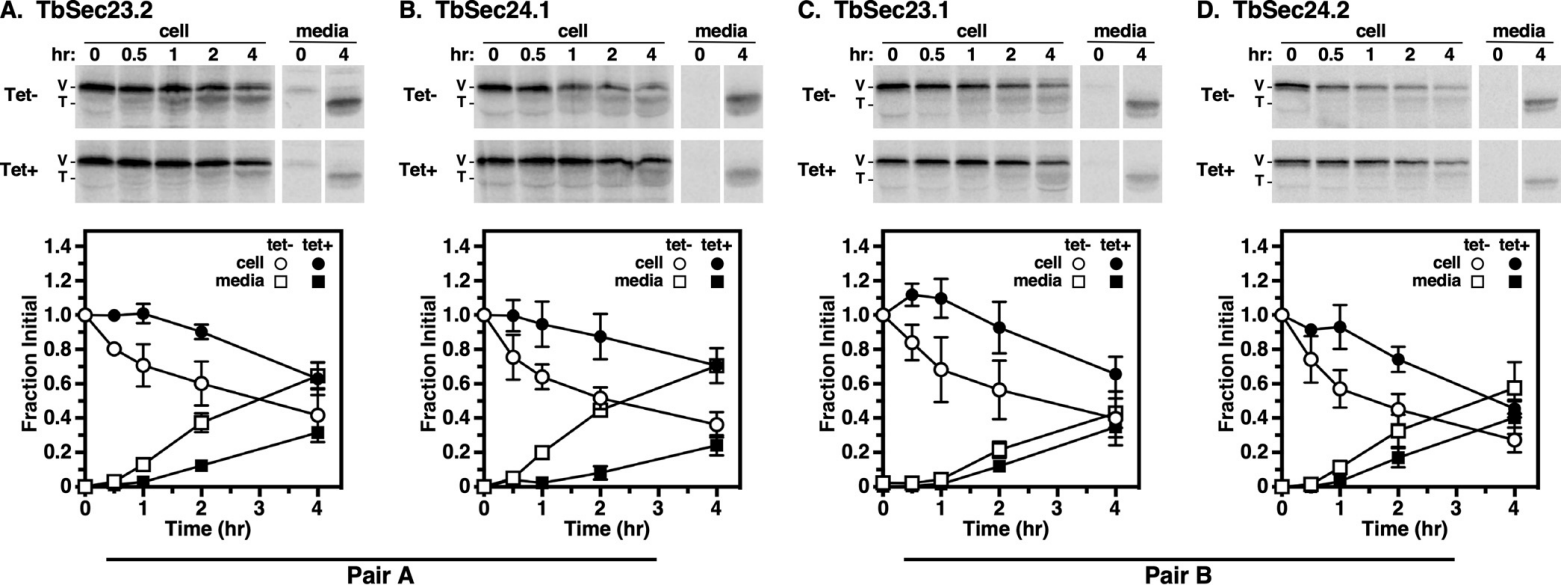
VSG transport in TbSec23/24 knockdowns. Specific dsRNA synthesis was induced for 48 h in TbSec23.2 (A), TbSec24.1 (B), TbSec23.1 (C), and TbSec24.2 (D) RNAi cell lines expressing VSG117. Pulse (15 min)/chase (4 h) radiolabeling was performed. CHX was added to block VSG synthesis, during the chase period. VSG was immunoprecipitated from cell lysates and media fractions at the indicated chase times and analyzed by SDS-PAGE and phosphorimaging (10^7^ cells/lane). (Top) Phosphorimages of representative matched gels from control (Tet^−^) and silenced (Tet^+^) cell lines. Mobilities of full-length (V) and truncated (T) VSG are indicated. Vertical white spaces indicate lanes that were excised post-image processing for the sake of presentation. Matched Tet^−^ and Tet^+^ gels are from the same processed phosphorimage. (Bottom) Quantification of loss of initial full-length VSG from cells with concomitant release of truncated VSG to the media. Three biological replicates are quantified, and the data are presented as mean ± SD.

The *VSG117* gene was introduced into each of the four PCF RNAi cell lines, and VSG transport was quantified as the rate of VSG loss from cells with the consequent appearance of truncated VSG in the medium. For reasons that are not fully apparent, initial assays using standard pulse/chase failed to achieve complete shutdown of protein radiolabeling during the initial phase of the chase period (Fig. S4). Oddly, this effect was also observed with other endogenous secretory reporters in these four VSG- expressing RNAi cell lines (unpublished data). Consequently, cycloheximide (CHX) was used to achieve a complete shutdown of protein synthesis (Fig. 4A to D). In each case, the knockdown of TbSec23 or TbSec24 subunits resulted in an ~3 to 4-fold delay in VSG transport from the ER to the cell surface. Precise half-times (*t*_1/2_) determined by nonlinear regression (Fig. S5) are reported in Table 1. These calculated halftimes likely underestimate the true rate of ER exit because the assay measures three consecutive processes: ER exit, transport to the cell surface, and proteolytic release to the media. Nevertheless, the differences between matched Tet^−^ and Tet^+^ data sets were statistically significant as indicated by nonoverlapping 95% CI ranges. Collectively, these data indicated that RNAi silencing of any of the COPII inner layer components had a similar negative effect on VSG transport. Thus, unlike in BSF, in PCF trypanosomes, there is a loss of GPI-dependent cargo specificity with respect to the inner layer of the COPII vesicles, with neither Pair A nor Pair B being favored.

**TABLE 1:** Kinetics of reporter transport

Reporter	Pair	RNAi target	Tet	*t*_1/2_ (hr)^*a*^	95% CI (hr)^*a*^	R^2^^*a*^
VSG^*b*^	A	TbSec23.2	−	2.7	2.1-3.5	0.77
			+	7.9	5.9-11.3	0.74
				
		TbSec24.1	−	2.1	1.7-2.7	0.82
			+	8.8	6.2-14.3	0.60
					
	B	TbSec23.1	−	2.5	1.9-3.5	0.73
			+	10.2	5.8-28.5	0.44
				
		TbSec24.2	−	1.6	1.3-2.1	0.85
			+	4.3	3.4-5.5	0.85
TbCatL^*c*^	A	TbSec23.2	−	0.6	0.5-0.8	0.89
			+	1.6	1.3-2.2	0.84
				
		TbSec24.1	−	0.7	0.6-1.0	0.88
			+	2.6	2.0-3.8	0.78
					
	B	TbSec23.1	−	0.9	0.7-1.0	0.94
			+	1.2	0.9-1.5	0.86
				
		TbSec24.2	−	0.6	0.5-0.8	0.92
			+	0.8	0.7-1.0	0.94
p67^*d*^	A	TbSec23.2	−	4.4	3.7-5.2	0.90
			+	10.1	6.7-17.2	0.56
				
		TbSec24.1	−	5.2	4.4-6.2	0.89
			+	11.5	8.8-16.0	0.73
					
	B	TbSec23.1	−	5.0	4.2-6.1	0.89
			+	15.0	9.5-30.2	0.46
				
		TbSec24.2	−	4.9	4.4-5.5	0.96
			+	10.6	8.6-13.4	0.80

a Halftimes and 95% confidence intervals (CI) were calculated by nonlinear regression (Fig. S5) and are presented in hrs. The degree of correlation between biological replicates is represented as R^2^. Halftimes generated by these analyses do not have error values. When comparing matched Tet^−/+^ data sets, any nonoverlap in 95% CI ranges have *P* ≤ 0.05 (70).

b VSG was measured as a loss of full-length VSG from cell fractions.

c TbCatL was measured as a loss of initial precursors (X+I).

d p67 was measured as a loss of initial gp100 ER glycoform.

10.1128/msphere.00188-22.4 FIG S4 VSG transport in TbSec23/24 knockdowns without cycloheximide (CHX). Specific dsRNA synthesis was induced for 48 h in TbSec23.2 (A), TbSec24.1 (B), TbSec23.1 (C), and TbSec24.2 (D) RNAi cell lines expressing VSG117. Pulse (15 min)/Chase (4 hrs) radiolabeling was performed without CHX. VSG was specifically immunoprecipitated from cell lysates and media fractions at the indicated chase times and analyzed by SDS-PAGE and phosphorimaging (10^7^ cells/lane). Loss of full-length VSG from cells (circle) with concomitant release of truncated VSG to the media (square) was quantified. One (N = 1), two (N = 2), or four (N = 4) biological replicates were quantified, and the data are presented as mean ± SD. Initial characterization of VSG transport indicated continued incorporation of [^35^S]methionine/cysteine labeling during the early period of the chase (0 to 30 mins), which obscures loss of full-length VSG from cell fraction. It is important to note that previous VSG trafficking studies in procyclic trypanosomes did not observe this effect (22). We believe that this effect is specific to the VSG-expressing cell lines such that after shut-off of incorporation by diluting with nonradioactive replete media there is an internal pool of radiolabeled aminoacyl tRNAs that transiently continues to be incorporated into newly synthesized proteins, leading to the observed increase in signal. To circumvent this problem, the trafficking of VSG was re-analyzed in the presence of CHX to block protein synthesis (Fig. 4). Download FIG S4, PDF file, 0.1 MB. Copyright © 2022 Sharif and Bangs. 2022 Sharif and Bangs. https://creativecommons.org/licenses/by/4.0/ This content is distributed under the terms of the Creative Commons Attribution 4.0 International license.

**FIG 5:**
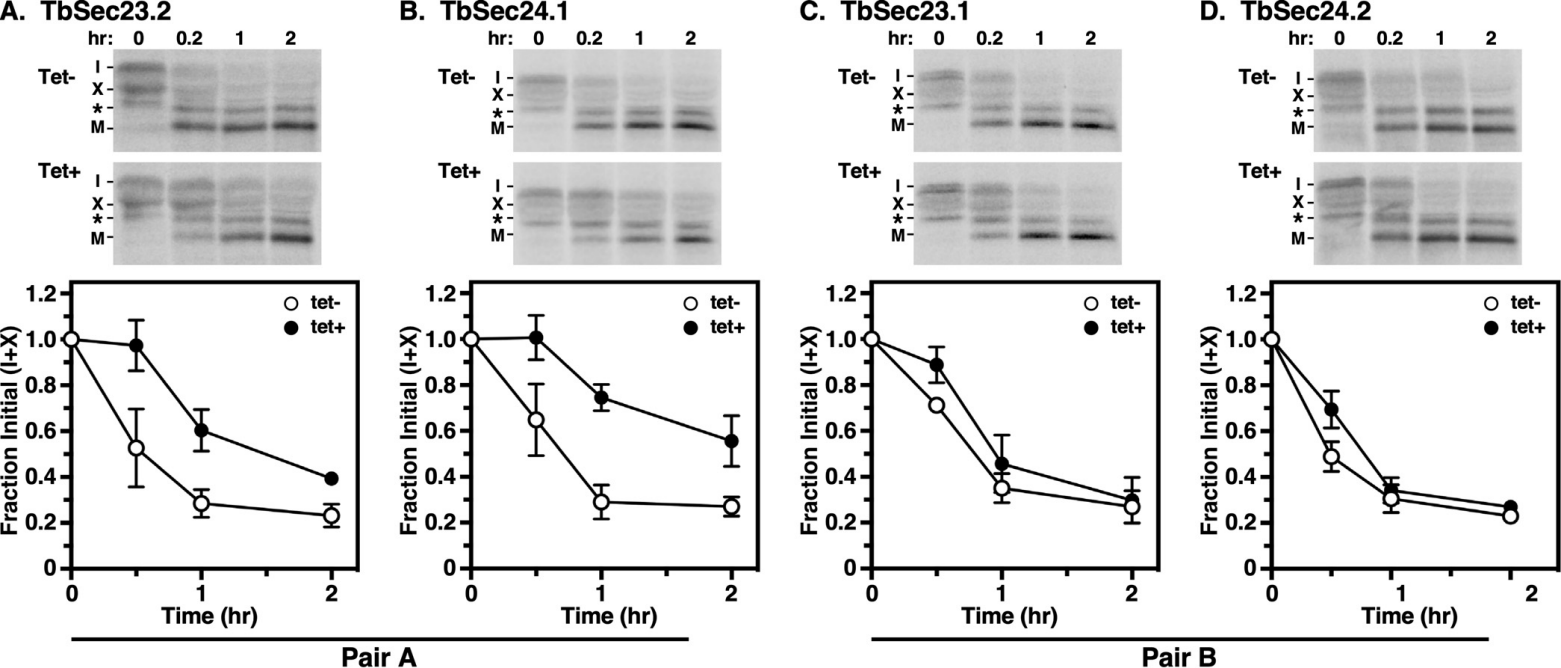
Transport of TbCatL in TbSec23/24 knockdowns. Specific dsRNA synthesis was induced for 48 h in TbSec23.2 (A), TbSec24.1 (B), TbSec23.1 (C), and TbSec24.2 (D) RNAi cell lines. Pulse (10 min)/Chase (2 h) radiolabeling was performed. TbCatL was immunoprecipitated from cell lysates at the indicated chase times and analyzed by SDS-PAGE and phosphorimaging (10^7^ cells/lane). (Top) Phosphorimages of representative matched gels from control (Tet^−^; upper) and silenced (Tet^+^; lower). Mobilities of initial precursors (I and X) and the lysosomal mature (M) form are indicated. Matched Tet^−^ and Tet^+^ gels are from the same processed phosphorimage. (Bottom) Quantification of loss of the initial precursors (I and X). The nonspecific band (*) was not included in the quantification. Three biological replicates were quantified, and the data are presented as mean ± SD.

**FIG 6:**
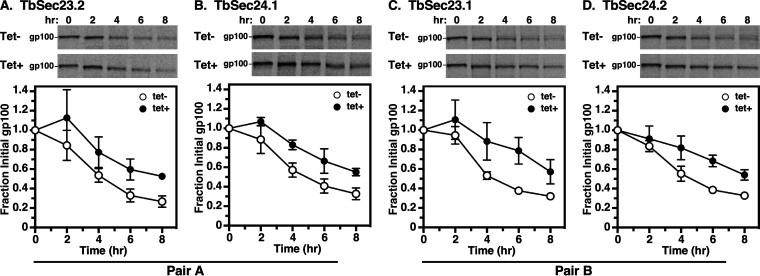
p67 transport in TbSec23/24 knockdowns. Specific dsRNA synthesis was induced for 48 h in TbSec23.2 (A), TbSec24.1 (B), TbSec23.1 (C), and TbSec24.2 (D) RNAi cell lines. Pulse (15 min)/Chase (8 h) radiolabeling was performed. p67 was immunoprecipitated from cell lysates at the indicated chase times and analyzed by SDS-PAGE and phosphorimaging (10^7^ cells/lane). (Top) Phosphorimages of representative matched gels from control (Tet^−^) and silenced (Tet^+^). Mobility of the gp100 precursor is indicated. Matched Tet^−^ and Tet^+^ gels are from the same processed phosphorimage. (Bottom) Quantification of loss of the initial ER precursor gp100. Three biological replicates were quantified, and the data are presented as mean ± SD.

10.1128/msphere.00188-22.5 FIG S5 Nonlinear regression models for reporter transport in TbSec23/24 knockdowns. Regression analysis of datasets in Fig. 4 (VSG; A to D), Fig. 5 (TbCatL; E to H), and Fig. 6 (p67; H to K). Kinetics of transport was quantified using nonlinear regression models (see Table 1) for control (Tet^−^) and postinduction with RNAi (Tet^+^) TbSec23 or TbSec24 subunits. Data analyses were performed in Prism 9 (GraphPad Software). In each graph, three biological replicates are quantified. The degree of correlation between each of the three biological replicates is reported in Table 1. The nonlinear graphs enabled the extrapolation of datasets and calculation of more process half-time of transport post RNAi silencing. Download FIG S5, PDF file, 0.2 MB. Copyright © 2022 Sharif and Bangs. 2022 Sharif and Bangs. https://creativecommons.org/licenses/by/4.0/ This content is distributed under the terms of the Creative Commons Attribution 4.0 International license.

### TbCatL transport is preferentially dependent on Pair A.

To better understand the changes in COPII cargo selectivity between the two stages, we analyzed the trafficking of endogenous cathepsin L (TbCatL), an endogenous soluble lysosomal hydrolase (40). TbCatL was initially synthesized in the ER as 53 (I) and 50 kDa (X) proproteins. The I and X proproteins were transported to the lysosome for proteolytic processing resulting in a single active mature form (M, 44 kDa). In these experiments, we observed these three bands for TbCatL along with a previously unseen 48 kDa extra band (indicated by *). Detection of this band was variable between experiments and quantitative analysis showed that band intensity did not decrease over time (Fig. S6A and B). In addition, band intensity did not increase over time with FMK024, an inhibitor that blocks proteolytic activation of TbCatL to the mature M form (Fig. S6C and D) (41, 42). Collectively these data indicate that the 48 kDa band is nonspecific and unrelated to the transport of TbCatL.

10.1128/msphere.00188-22.6 FIG S6 TbCatL experiments and observation of the nonspecific band. Specific dsRNA synthesis was induced for 48 h in TbSec23.2 (A) and TbSec23.1 (B) RNAi cell lines. Pulse (10 min)/chase (2 hrs) radiolabeling was performed. TbCatL was specifically immunoprecipitated from cell lysates at the indicated chase times and analyzed by SDS-PAGE and phosphorimaging (10^7^ cells/lane). Expression of the nonspecific band (*) over time was quantified. Three biological replicates were quantified, and the data are presented as mean ± SD. Detection of the nonspecific band was variable; the band was detected 87.1% (27/31 experiments) of the time (unpublished data). Furthermore, the band intensity did not decrease over time in both controls (Tet^−^; open circles) and silenced (Tet^+^; closed circles). (C) Specific dsRNA synthesis was induced for 48 h in the TbSec23.1 RNAi cell line. Pulse (10 min)/chase (2 hrs) radiolabeling was performed. FMK024, a selective thiol protease inhibitor, was added to block proteolytic activation of the mature M form. TbCatL was specifically immunoprecipitated from cell lysates at the indicated chase times and analyzed by SDS-PAGE and phosphorimaging (10^7^ cells/lane). Expression of the nonspecific band (*) over time was quantified. Three biological replicates are quantified, and the data are presented as mean ± SD. Treatment with FMK024 did not increase nonspecific band intensity over time in both controls (Tet^−^; open circles) and silenced (Tet^+^; closed circles). (D) TbSec23.1 cell line’s phosphorimages of representative matched gels from control (FMK^−^; upper) and treated (FMK+; lower) in the noninduced (Tet^−^) condition. Matched FMK^−^ and FMK^+^ gels are from the same processed phosphorimage. Mobilities of initial precursors (I and X), the nonspecific band (*) and the lysosomal mature (M) form are indicated. Without inhibition, initial precursors (I + X) are converted to the mature M form while the nonspecific band (*) remains relatively the same over time. With inhibition, we observed that the initial precursors and the nonspecific band remain the same over time. Collectively these data indicate that the nonspecific band is not part of the TbCatL transport-dependent processing dynamic. Download FIG S6, PDF file, 0.2 MB. Copyright © 2022 Sharif and Bangs. 2022 Sharif and Bangs. https://creativecommons.org/licenses/by/4.0/ This content is distributed under the terms of the Creative Commons Attribution 4.0 International license.

Therefore, to determine the roles of TbSec23 and Sec24 subunits in TbCatL trafficking from the ER to the lysosomes, we quantified the loss of initial precursors (I+X) upon transport to the lysosome. Knockdown of either Pair A subunit resulted in statistically significant delays (~3 to 4-fold) in TbCatL transport to the lysosome (Fig. 5A and B; Table 1). However, knockdown of either Pair B subunit had a minimal effect on TbCatL transport (Fig. 5C and D; Table 1). These results differ from BSF trypanosomes, where independent RNAi silencing of Pair A or Pair B components had minimal effects on the trafficking of the soluble TbCatL cargo (35). Collectively, these findings support a model in which there is a stage-specific selectivity in PCF cells via Pair A in the transport of TbCatL that was not found in the BSF trypanosomes.

### Pairs A and B are required for efficient p67 transport.

Finally, we examined the trafficking of p67, a lysosomal-associated type I membrane glycoprotein in BSF trypanosomes (38). In both stages, p67 is initially synthesized in the ER as 100 kDa N-glycosylated protein (gp100). In BSF trypanosomes, post-ER exit, N-glycan modification in the Golgi converts gp100 to a 150 kDa glycoform (gp150). Thereafter, it is transported to the lysosome, where proteolytic fragmentation generates smaller quasi-stable 42 kDa, and 32 kDa glycoforms. In PCF trypanosomes, gp100 is trafficked to the lysosome without N-glycan processing in the Golgi, but the proteolytic generation of gp42 and gp32 glycoforms still occurs. Thus, while there are several stage-specific aspects to the processing of p67, loss of the gp100 glycoform is a valid metric for trafficking p67 from the ER. Knockdown of all Pair A and Pair B subunits resulted in statistically significant delays (2 to 3-fold) in the disappearance of gp100 (Fig. 6A to D; Table 1). These data supported a model in which both Pair A and B were required for the efficient p67 transport from the ER to the lysosome. These findings differ from BSF trypanosomes, where independent RNAi knockdown of Pair A or Pair B components resulted in a minor defect in the trafficking of p67 cargo (35).

## DISCUSSION

Trypanosomes are unique in that they have just one or two ERES closely juxtaposed to corresponding Golgi clusters. In addition, they have two orthologues each of Sec23 and Sec24 that form obligate, exclusive, and essential heterodimers, Pair A (TbSec23.1 and TbSec23.2) and Pair B (TbSec24.1 and TbSec24.2) (35). RNAi studies in BSF trypanosomes indicated that these Pairs are redundant for forwarding trafficking of soluble (TbCatL) and transmembrane (p67) cargos from the ER. However, loss of either component of Pair A, but not Pair B, drastically delayed GPI-APs (4 to 5-fold). GPI anchors are forward signals in trypanosomes (18, 19), and the Pair A dependence of GPI-AP ER exit is mediated by the BSF-specific cohort of p24 proteins (TbERP1,2,3,8) (21). These data indicate that COPII selectively mediates GPI-dependent ER exit through Pair A in BSF trypanosomes.

We have now addressed the role of the COPII inner layer in transporting secretory cargos from the ERES in PCF trypanosomes. Unlike in BSF trypanosomes, independent RNAi knockdown of TbSec23/24 subunits was not immediately lethal, but all subunits are essential for normal growth. The failure to achieve complete lethality is likely due to the slower replication and reduced cargo load of PCF cells. Both TbSec23 subunits colocalize with TbSec24.1, and by extension TbSec24.2, to the single ERES in interphase cells, consistent with the colocalization of all TbSec23/24 subunits in BSF, and the localization of both TbSec24 subunits in PCF trypanosomes (35, 36). However, unlike in BSF cells, knockdown of each subunit of Pair A and B reduces the rate of ER exit for GPI-APs 3 to 5-fold. Thus, these Sec23/Sec24 heterodimers are both responsible for GPI-anchored cargo in PCF trypanosomes. These results suggest a model in which COPII-specific trafficking of GPI-APs is a stage-specific phenomenon found only in BSF trypanosomes.

Given these stage-specific differences in GPI-dependent cargo, we investigated both transmembrane and soluble cargos previously examined in BSF trypanosomes, p67 and TbCatL, respectively. p67 is a lysosomal Type I transmembrane glycoprotein, which presumably does not require p24 receptors for COPII loading. Likely, p67 leaves the ER by bulk flow as deletion of the cytoplasmic domain does not affect its rate of forwarding trafficking (38). Silencing of either Pair A or B in BSF cells had minimal effect on p67 transport indicating that they are functionally redundant for this cargo (35). In contrast, in PCF parasites, independent knockdown of either Pair A or B subunits resulted in a 2 to 3-fold delay in lysosomal trafficking of p67. Thus, both Pair A and B are required for efficient ER exit of p67. TbCatL is a soluble lysosomal protease (42, 43). In BSF trypanosomes, silencing of TbSec23 or TbSec24 subunits had minimal effect on TbCatL trafficking (35). However, delivery to the lysosome was dependent on both Sar1 and all four expressed p24 orthologues (TbERP1,2,3,8) (21, 35), indicating that ER exit is indeed dependent on the COPII machinery. Consequently, it appears that Pairs A and B are functionally redundant for this cargo in BSF trypanosomes. In contrast, in PCF trypanosomes Pair A selectively mediates ER exit of TbCatL. Silencing reduces lysosomal delivery 3 to 4-fold, whereas silencing of Pair B has no effect. Collectively, these data indicate that there are stage-specific differences in COPII-mediated cargo selection in African trypanosomes (discussed below).

There are substantial differences in COPII machinery and COPII-dependent ER exit of cargo within eukaryotic cells. For instance, while S. cerevisiae encodes one paralogue of *Sec23* (*Sec23p*), there are two paralogues of *Sec23* (*Sec23A* and *Sec23B*) in mammals (44, 45). For the cargo-capturing subunit Sec24, there are three paralogues in yeast (Sec24p, Lst1p, and Iss1p) and four in mammalians (Sec24A-D) (27). Unlike in trypanosomes, not all COPII paralogues are required for viability in these systems. For example, all three Sec24 paralogues in S. cerevisiae interact with Sec23p, but only Sec24p is essential (46). However, while depletion of Lst1p is not lethal, it does selectively inhibit transport of the plasma membrane proton-ATPase (Pma1p) (47). In mammalian cells, the two paralogues of Sec23 interact interchangeably with all four paralogues of Sec24, and none are essential for viability (48). However, the expression of Sec23A and Sec23B is tissue-specific, and deficiencies in either resulted in distinct pathologies.

Another difference between yeast and mammals is the number of ERES. In yeast, where there are tens of ERES (49, 50), GPI-APs are segregated to a subset defined by ceramide-enriched membranes (51, 52), and loaded into COPII vesicles with a distinct subunit composition (Sec23p/Lst1) compared to vesicles (Sec23p/Sec24p) carrying non-GPI cargoes at different ERES. The GPI anchor structure controls cargo selection in yeast in two ways (reviewed in reference (53)). First, GPI lipid remodeling in the ER leads to the clustering and sorting of ceramide-rich ERES membranes. Second, GPI glycan remodeling, which involves the removal of ethanolamine phosphate (EtNP) moieties from the glycan core, allows interaction between the GPI-anchor and p24 cargo receptors. This in turn stimulates recruitment of the Sec23p/Lst1 heterodimers via cytoplasmic interaction with Lst1. In contrast, in mammalian cells, which have hundreds of ERES (54, 55), GPI-APs are not segregated and sorted from other secretory proteins at ERES. However, while lipid remodeling does not occur in mammalian cells, ER exit of GPI-APs is still contingent on GPI-glycan remodeling (removal of EtNP), recognition by p24 receptors, and loading into COPII vesicles via interaction with Sec24C or Sec24D subunits (53).

The situation in trypanosomes contrasts remarkably with yeast and mammals. First, all Sec23 and Sec24 subunits are essential. Second, having only one or two ERES (35, 36), ER cargo cannot be segregated via distinct ERES nor can sorting be lipid-based because GPI lipid remodeling does not occur, and blocking ceramide synthesis does not affect the trafficking of GPI-APs (56). In addition, the stage-specific differences in GPI-selectivity cannot be attributed to differences in COPII vesicle composition since Pair A and B are constitutively expressed (35, 57). Finally, the same core undecorated GPI glycan structure (no EtNP) is attached to the protein in both BSF and PCF trypanosomes (58). Variable core processing does occur in each stage, but this happens following transport to the Golgi (40, 59, 60).

A possible explanation for the observed stage-specific differences in COPII GPI-AP selectivity is differential expression of the oligomeric p24 receptors that bind Sec24 during cargo loading. These receptors have motifs in their short cytoplasmic domains for binding Sec24 in the Sar1:Sec23:Sec24 prebudding complex, and lumenal α-helical domains that mediate oligomerization (53). BSF and PCF trypanosomes each express a different cohort (BSF: TbERP1, 2, 3, and 8; PCF: TbERP1, 2, 4, and 8) (21). All four of these p24s are required for efficient ER exit of GPI-APs in BSF trypanosomes. In PCF studies, only TbERP4 has been examined, and it too is required for efficient transport of GPI-APs from the ER. Interestingly, the two stage-specific p24s, TbERP3, and TbERP4, are 54% identical at the amino acid level, and notably are 9 of 10 identical in the cytoplasmic domains, the sole difference being Arg197 in TbERP3 and Cys195 in TbERP4. Perhaps this difference is sufficient to alter the binding of TbSec24 subunits in the prebudding complex from selective for Pair A in BSF to permissive for either Pair A or Pair B in PCF cells. Alternatively, differences in the α-helical domains may result in altered receptor stoichiometry that in turn affects the recognition of TbSec24s on the cytoplasmic side of budding COPII vesicles. It should also be noted that while we were unable to detect TbERP4 expression in BSF cells in our original characterization of trypanosomal p24s (21), low levels of expression were detected in a recent proteomic study (57). In contrast, TbERP3 was barely detectable in PCF cells in the same study. These factors may subtly influence either of the possible explanations presented above.

A similar scenario can be invoked to account for stage-specific differences in ER exit of TbCatL. In this case, the p24 complex responsible for ER exit in BSF cells is recognized by both Pair A and Pair B, while in PCF cells it is recognized only by Pair A. Again, we would predict that this distinction is due to differential expression of TbERP3 and TbERP4, and consequently in the p24 complexes formed in each stage. Finally, the behavior of p67 in BSF versus PCF cells is simpler to explain as it is likely that p67 leaves the ER by bulk flow (38). Consequently, its ER exit is dependent on the basal rate of COPII vesicle formation, which must be higher in BSF cells as expression of all TbSec23 and TbSec24 subunits is 2 to 5-fold higher in these stages (57). Thus, in the absence of either Pair A or Pair B, there is sufficient capacity to maintain normal levels of p67 transport in BSF cells. Conversely, in PCF cells with lower capacity, loss of p67 reduces the overall rate of ER exit.

The insights of Demmel et al. (36) on TbSec24s in PCF trypanosomes add additional complexity to our understanding of COPII function in trypanosomes. Knockdown of TbSec24.1 or TbSec24.2 each reduced secretion of BiPN, the soluble globular ATPase domain of BiP, and a bulk flow reporter (61), 2 to 3-fold, consistent with our results with p67. Likewise, the Golgi glycosyltransferase TbGntB was partially mislocalized to the ER suggesting that both Pair A and Pair B can facilitate its transport. Most striking were the phenotypes of the Golgi matrix proteins TbGRASP and TbGolgin63. Knockdown of TbSec24.1 (Pair A) specifically altered the localization of these reporters to the ER. TbGRASP is most closely related to mammalian GRASPs, which associate with membranes via N-terminal myristoylation. The localization of these proteins to the Golgi is direct and independent of the COPII machinery (62). Thus, it is likely that TbSec24.1 mediates ER exit of some other protein that specifies Golgi localization of TbGRASP. On the other hand, TbGolgin63 is a tail-anchored protein with a transmembrane domain at the extreme C terminus. These proteins are specifically targeted to ER membranes following synthesis and can then be exported from the ER by COPII vesicles, e.g., vesicle SNARE proteins (63). As proposed by Demmel et al. (36), we would envision a direct and specific interaction of TbGolgin63 with TbSec24.1.

Collectively these data expand our knowledge of protein trafficking in the early secretory pathway of trypanosomes, which differ markedly in this regard from other eukaryotic model systems. However, many questions remain. For instance, nothing is known about the mixing of distinct Sec23:Sec24 heterodimers in COPII vesicles in any system. One question that arises in trypanosomes is whether Pair A and Pair B form a homotypic class of vesicle or if they are found in a single heterotypic carrier. Recent structural studies indicate that the inner layer of COPII vesicles is densely packed with Sar1:Sec23:Sec24 protomers that interact laterally in Sec23:Sec23 contacts (64). TbSec23.1 and TbSec24.2 are 70% similar. Is this enough, especially in the critical contact sites, to allow the formation of mixed vesicles? In addition, unlike the other model systems trypanosomes have two paralogues of Sec13 (TbSec13.1, Tb927.10.14180; TbSec13.2, Tb927.11.8120) that with Sec31 form the outer layer of the COPII coat. Sec13 is thought to give rigidity to the rod-like heterotetramer to aid in membrane deformation (65), but itself does not interact with the inner layer. How might these paralogues influence cargo selection in trypanosomes? These questions and others suggest that trypanosomes have much to offer in defining basic secretory processes in all eukaryotes.

## MATERIALS AND METHODS

### Maintenance of trypanosomes.

All experiments were performed with T. brucei *brucei* Lister strain 427 procyclic (Pro-1) cell line or tetracycline responsive procyclic form (29-13) cell line (66). All cell lines were cultured in Cunningham's medium supplemented with 10% tetracycline-free fetal bovine serum (Atlanta Biologicals, Lawrenceville, GA) at 27°C (67). All experiments were performed with cells harvested at the mid-to-late log phase (0.5 × 10^7^ to 1 × 10^7^ cells/mL).

### Construction of inducible cell lines.

The assembly of all four TbSec23/24 RNAi constructs has been described previously (35). In short, for each construct, a segment (~1 to 2 Kbps) from the start of each open reading frame (ORF) was cloned into an inducible double-stranded RNA (dsRNA) vector, p2T7Ti (68). Inserts were *TbSec23.1* (Tb927.8.3660, nt 18 to 2101), *TbSec23.2* (Tb927.10.7740, nt 9 to 1724), *TbSec24.1* (Tb927.3.1210, nt 17 to 1093), and *TbSec24.2* (Tb927.3.5420, nt 171 to 2101). The constructs were linearized with NotI and transfected independently into the 29-13 PCF cell line. Positive transformants were selected with phleomycin, and clonal populations were obtained by limiting dilution. As an additional step, genomic insertion of the RNAi construct was confirmed by PCR (Fig. S1B and C). For this cell lines were tested using a forward primer (FR: 5′-CTATCGATGTATGCCTTGGCC-3′) targeting a common upstream region on the inserted vector's backbone and four specific reverse primers: TbSec23.1 (RP: 5′-ATTCCCGAGCTCCGCAGT-3′), TbSec23.2 (RP: 5′-TTTCTCCCGCGTTGTTTCTACG-3′), TbSec24.1 (RP: 5′-GGAAACACACGGAACCTCTT-3′), and TbSec24.2 (RP: 5′-CTACTGAACAATGTGTCAACTCGGG-3′). Cloning of the VSG117 reporter into the pXS2 vector has been previously described (18). The VSG117 gene was digested from the pXS2 vector and cloned into a pXS5 vector (a derivative of pXS2 (38)) with a puromycin resistance cassette. The vector was linearized with XhoI and transfected into the four TbSec23/24 RNAi cell lines. Positive transformants were selected with puromycin, and clonal populations were obtained by limiting dilution. VSG-positive clones were confirmed by Western blotting (Fig. S3).

### Construction of epitope-tagged cell lines.

For localization assays, TbSec24.2:Ty, TbSec23.2:HA, and TbSec23.1:HA *in situ* tagging constructs were used. These constructs have been described previously (35). All three constructs were linearized with KpnI/SacI. Using the Pro-1 cell line, we first transfected with the TbSec24.2:Ty construct. Clonal populations were obtained by limiting dilution and hygromycin drug selection. TbSec24.2:Ty positive cell lines were screened with Western blot. Using the TbSec24.2:Ty cell line, we independently cotransfected TbSec23.1:HA or TbSec23.2:HA constructs. In both cases, positive transformants were selected with neomycin, and clonal populations were obtained by limiting dilution. TbSec23.1:HA/TbSec24.2:Ty and TbSec23.2:HA/TbSec24.2:Ty positive cell lines were confirmed with Western blot.

### RNA extraction and qRT-PCR.

Transcript levels of endogenous TbSec23/24 genes were determined using quantitative reverse transcription-PCR (qRT-PCR). Total RNA was isolated using RNeasy Minikit (Qiagen, Valencia, CA, USA). RNA was treated with DNAse1 on-column using RNase-Free DNase (Qiagen, Valencia, CA, USA), and cDNA was prepared using the iScript cDNA synthesis kit (Bio-Rad, Hercules, CA, USA) per manufacturer's instructions. qRT-PCRs were prepared using Power SYBR Green PCR Master Mix (Life Technologies, Carlsbad, CA, USA), diluted cDNAs, and specific primers targeting endogenous TbSec23.2 (FP: 5′-CTGGATAGTGCTGCGATTCA-3′ and RP: 5′-GCTCAGCATACCCTGCTTTC-3′), TbSec24.1 (FP: 5′-GAGACCGGTGACTGCGTTAT-3′ and RP: 5′-GTGCCTGCTCATCACAAAGA-3′), TbSec23.1 (FP: 5′-CGTCCGTGCTTCACCTTATT-3′ and RP: 5′-TGTCGCTTGAATGTCGACTC-3′), or TbSec24.2 (FP: 5′-ATGGTCAACGTGGTGGGTAT-3′ and RP: 5′-ATAGGCGTCGAAGTCGAGAA-3′). The qRT-PCRs were performed in the StepOne™ real-time PCR system (Life Technologies, Carlsbad, CA, USA). Each reaction was performed in triplicates, and for each transcript, melting curves indicated a single dominant product after amplification. Experimental transcripts were independently normalized to the internal reference gene *TbZFP3* (69). Three biological replicates were performed for each TbSec23/24 subunit and mean ± SD were quantified.

### Antibody, secondary, and blotting reagents.

Rabbit anti-VSG117, rabbit anti-TbCatL, rabbit anti-BiP, and mouse monoclonal anti-p67 were described previously (37, 38, 61). The rabbit anti-HA tag was purchased from Sigma-Aldrich (St. Louis, MO, USA). The mouse monoclonal anti-Ty was generated by Convance Laboratories Inc. (Denver, PA, USA).

### Immunofluorescence microscopy.

PCF trypanosome immunofluorescence staining was done as described previously with minor alterations (42). In short, 1 × 10^7^ cells were harvested, washed, and resuspended in 1 mL ice-cold 1× PBSG (PBS with 10 mg/mL glucose). One hundred microliters of cells (1 × 10^7^ cells/mL) were loaded onto SuperFrost Plus microscopy slides (VWR International, Radnor, PA, USA) in individual wells. Cells were fixed on the slides with the addition of 100 μL of fixing solution (PBS, 4% formaldehyde) for 30 min. Next, cells were washed with 1× PBS and 100 μL of permeabilization solution (PBS, 0.5% NP-40) was added to each well for 30 min. Cells were then washed with PBS and incubated with 100 μL of blocking solution (PBS, 10% normal goat serum, 0.1% NP-40) for 30 min. Cells were washed with PBS and stained with specific primary antibodies diluted in a blocking solution (100 μL) for 1 h. After primary antibody staining, cells were washed and stained with appropriate Alexa488- or Alexa594-conjugated secondary antibodies (Molecular Probes, Eugene, OR) diluted in blocking buffer (100 μL) for 30 min. Finally, slides were washed and mounted with DAPI fluoromount-G (Southern Biotech, Birmingham, AL, USA) for visualization. Serial 0.2 μm image Z-stacks were collected with capture time from 100 to 400 ms (100× PlanApo, oil immersion, 1.46 na) on a motorized Zeiss Axioimager M2 stand equipped with a rear-mounted excitation filter wheel, a triple pass (DAPI/FITC/Texas Red) emission cube, differential interference contrast optics. All images were captured with an Orca AG CCD camera (Hamamatsu, Bridgewater, NJ, USA) in Volocity 6.0 acquisition software (Improvision, Lexington, MA, USA). Postimaging individual channel stacks were deconvolved by a constrained iterative algorithm and merged using Volocity 6.0 restoration software.

### Pulse/chase transport analyses.

Pulse/chase metabolic radiolabeling with [^35^S]methionine/cysteine (Perkin Elmer, Waltham, MA, USA) and subsequent immunoprecipitation of radiolabeled proteins (VSG, TbCatL, and p67) from lysates were performed as previously described with minor alterations (42). In short, log-phase cells were harvested, washed with HEPES-buffered saline (HBS: 50 mM HepesKOH, pH 7.5, 50 mM NaCl, 5 mM KCl, 70 mM glucose), and resuspended in methionine/cysteine-minus labeling media (10^8^/mL, 15 min, 27°C). Labeling was initiated by the addition of [^35^S]methionine/cysteine (200 μC/mL, PerkinElmer, Waltham, MA); pulse times were 15 min for VSG, 10 min for TbCatL, and 15 min for p67. The chase period was initiated by 10-fold dilution with a complete medium, and samples were selected at specific time points as indicated in the relevant figures. Samples were separated into cell and media fractions; cells were lysed in radioimmunoprecipitation assay buffer (RIPA: 50 mM Tris-HCl, pH 8.0, 150 mM NaCl, 1.0% NP-40, 0.5% deoxycholate, and 0.1% SDS) and medium was supplemented with RIPA detergents. Immunoprecipitated proteins were analyzed by SDS-PAGE and phosphorimaging using a Typhoon FLA 9000 with native ImageQuant Software (GE Healthcare, Piscataway, NJ, USA). For some VSG pulse/chase experiments, as indicated in relevant figures, cycloheximide (CHX: 10 mg/mL) was introduced at the start of the chase period. In addition, for some TbCatL experiments, the lysosomal thiol protease inhibitor FMK024 (20 μM; MP Biomedicals, Aurora, OH) was introduced at the start of the chase period.

### Data analyses.

Phosphorimages were quantified using ImageJ (http://imagej.nih.gov/ij/). The intensities of specific bands (identical specific areas) within each lane were measured and corrected for background by subtracting the signal from an equivalent unlabeled area within the same lane. All subsequent data analysis was performed in Prism 9 (GraphPad Software Inc., San Diego, CA, USA).
