# Supplementary figures and images for: Stage-Specific COPII-Mediated Cargo Selectivity in African Trypanosomes
Source: mSphere. 2022 Jun 21;7(4):e00188-22. doi: 10.1128/msphere.00188-22 (PMC9429938; doi:10.1128/msphere.00188-22)

**A.**

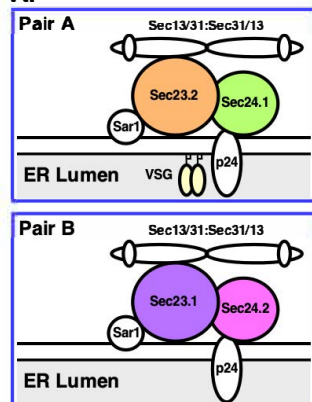

**B.**

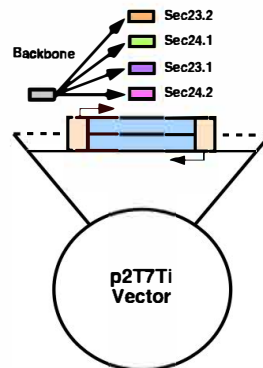

**C.**

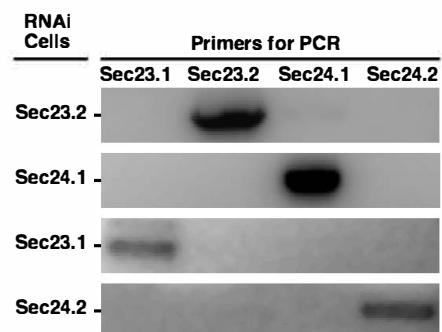

Supplement: FIG S1 [file msphere.00188-22-s0001.pdf]

**A. TbSec23.1**

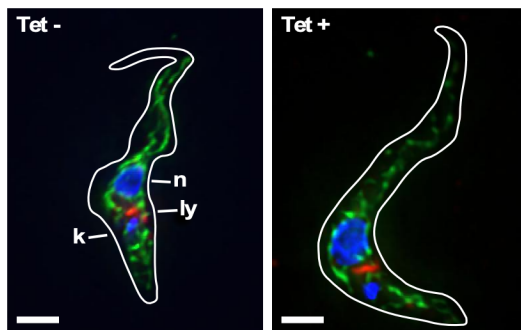

**C. TbSec24.1**

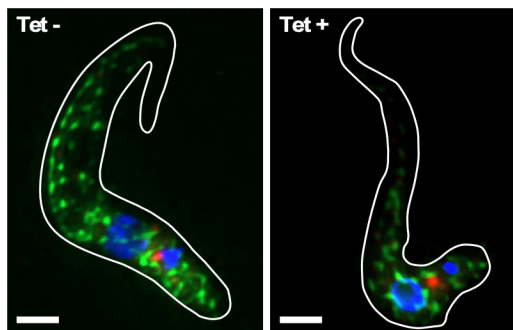

**B. TbSec23.2**

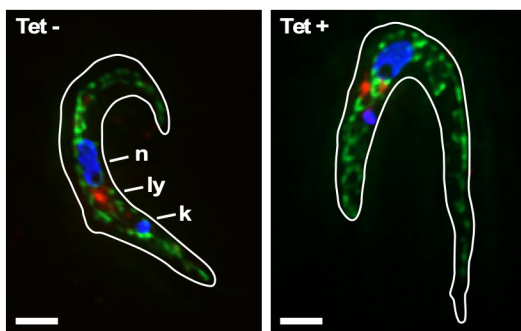

**D. TbSec24.2**

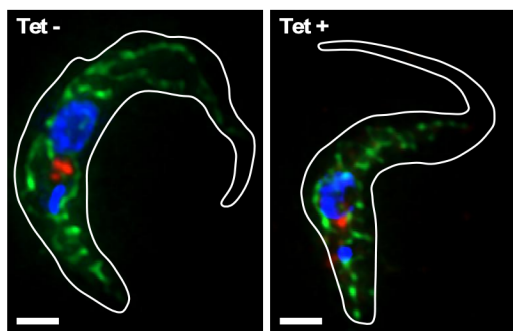

Supplement: FIG S2 [file msphere.00188-22-s0002.pdf]

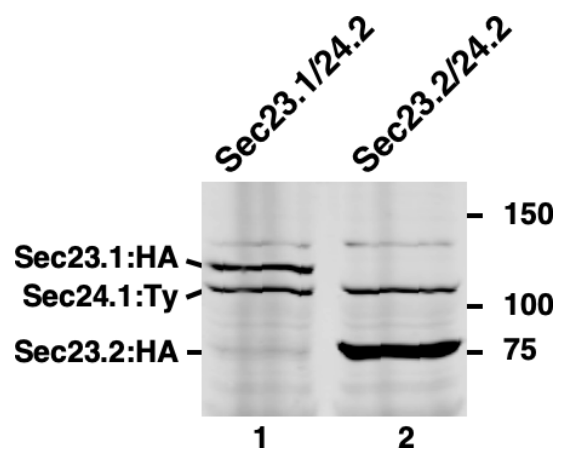

Supplement: FIG S3 [file msphere.00188-22-s0003.pdf]

A. TbSec23.2

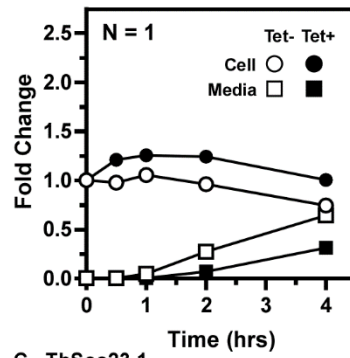

B. TbSec24.1

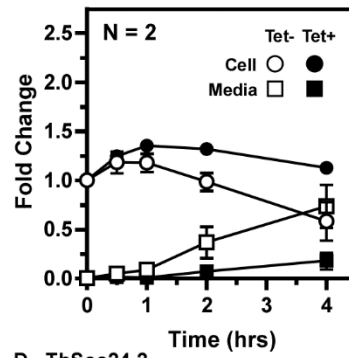

C. TbSec23.1

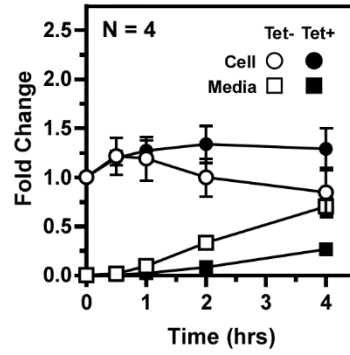

D. TbSec24.2

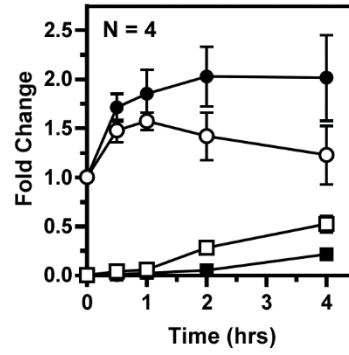

Supplement: FIG S4 [file msphere.00188-22-s0004.pdf]

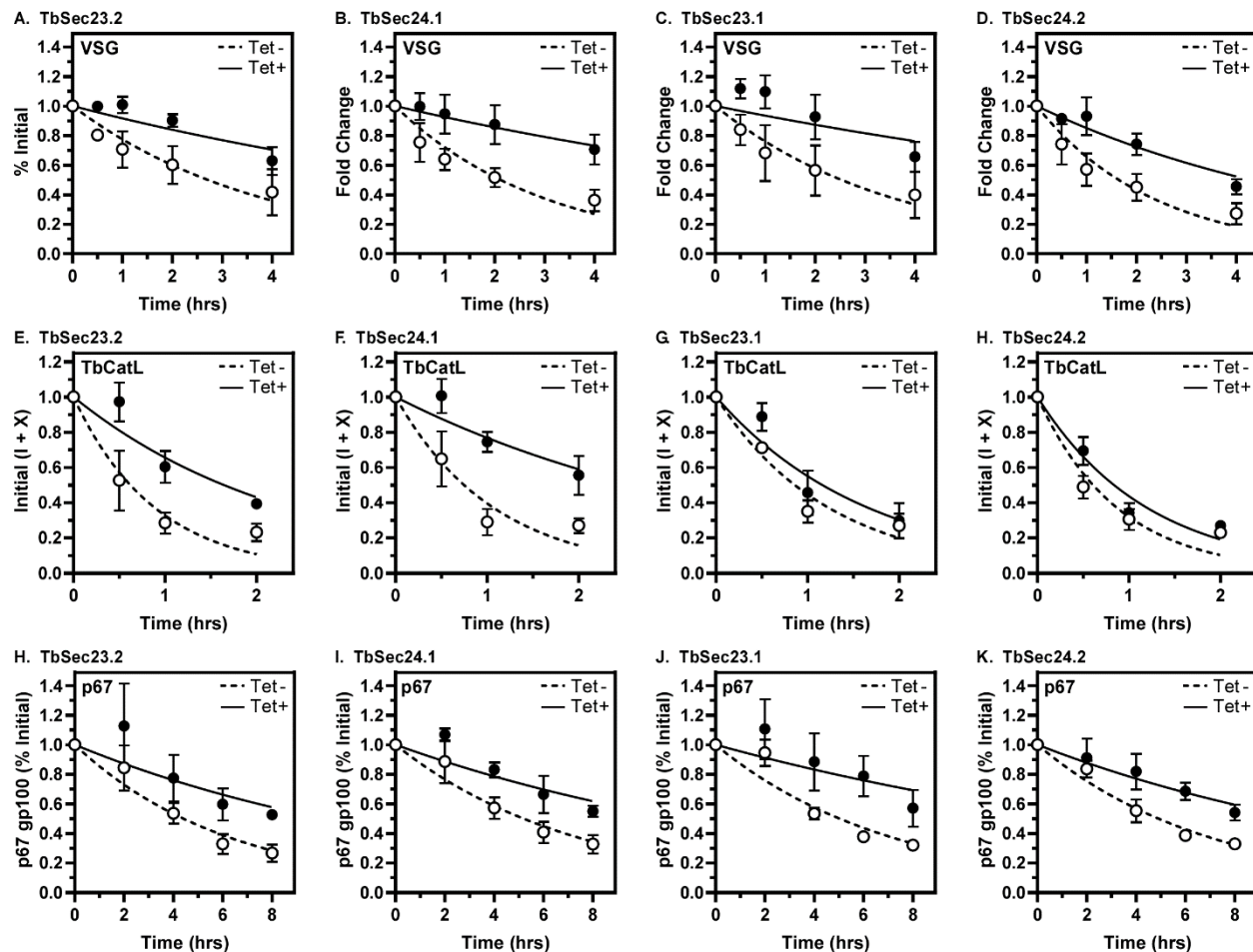

Supplement: FIG S5 [file msphere.00188-22-s0005.pdf]

A. TbSec23.2

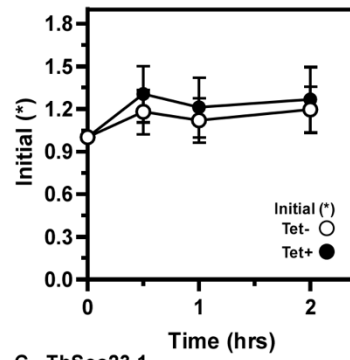

B. TbSec23.1

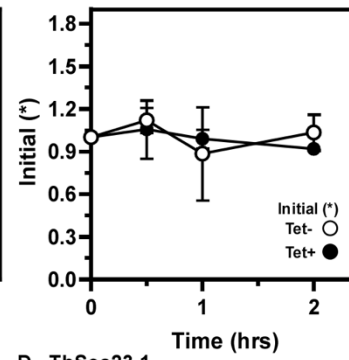

C. TbSec23.1

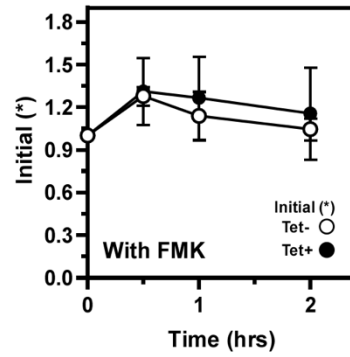

D. TbSec23.1

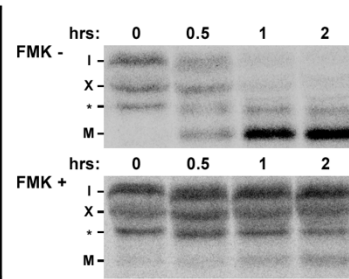

Supplement: FIG S6 [file msphere.00188-22-s0006.pdf]
